# Becoming a Researcher: An Empirical Study on the Factors Influencing Chinese PhD Students’ Research Career Intentions

**DOI:** 10.3390/bs15020123

**Published:** 2025-01-24

**Authors:** Xingqi Luo, Jie Yin, Yang Zou, Xianwei Liu, Wanli Ma, Yichu Deng

**Affiliations:** 1 College of Marxism, Xi’an University of Technology, Xi’an 710054, China; 1211111005@stu.xaut.edu.cn (X.L.); yinjie@xaut.edu.cn (J.Y.); 2 School of Electronic and Electrical Engineering, Baoji University of Arts and Sciences, Baoji 721016, China; 3 College of Business Administration, Capital University of Economics and Business, Beijing 100070, China; 4 School of Public Administration, Beihang University, Beijing 100191, China; 5 Faculty of Education, Henan University, Kaifeng 475001, China; mawl@buaa.edu.cn; 6 College of Holistic Education, Beijing University of Technology, Beijing 100124, China; dengyichu@bjut.edu.cn

**Keywords:** Chinese doctoral students, research career intentions, proactive personality, employability social capital, career adaptability, gender, career construction theory

## Abstract

Increasing the attractiveness of research careers for PhD students has been a key objective of science, technology, and innovation policies worldwide. This study draws on an expanded career construction theory framework to examine the effects of proactive personality, employability social capital, and career adaptability on PhD students’ research career intentions as well as the moderating role of gender in this structural model. We collected data from 795 Chinese PhD students at 10 universities via an online questionnaire survey. Structural equation modeling analysis revealed that proactive personality had no significant direct effect on research career intentions; however, it could influence research career intentions indirectly via the separate and chain mediating effects of employability social capital and career adaptability. In addition, gender was shown to moderate the relationship between employability social capital and research career intentions. The theoretical and practical implications of this research are highlighted, and recommendations for future research are discussed.

## 1. Introduction

“Research career is defined as a trajectory of research-based employment that can take place within and across academia, research councils and research institutes, industry, government laboratories and agencies” (Thomson & Gooberman-Hill, 2024, p. 34). A significant subgroup of researchers consists of PhD holders, who have completed highly specialized training in research and are expected to make original contributions to the advancement of science, technology, engineering, and societal knowledge (Auriol et al., 2016; Dorenkamp & Weiß, 2018). Therefore, earning a PhD is viewed both as a taken-for-granted pathway toward a research career and an opportunity to fulfill one’s own scientific and research interests and curiosities (Bin et al., 2016). However, during the past several decades, the number of PhD candidates and holders who considered quitting the research profession completely increased dramatically, especially in countries featuring long-developed histories of PhD education, such as the USA and several European countries, where on average 50% of doctorate holders work in non-research careers (Auriol et al., 2013). Although research careers remain the main choice for PhD students in China, a recent study based on national data concerning employment outcomes among PhD graduates from 2015 to 2020 revealed that more than 30% of doctorate holders in China leave the field of research (H. Luo et al., 2022), despite the multitude of research-related positions that are available to them in the business sector (Shen et al., 2018).

Although increasing the attractiveness of research careers has been a key objective of science, technology, and innovation policies in China and other countries featuring developed doctoral education systems (Foubert et al., 2020; Hancock, 2021), empirical research on doctoral employment has only a short history, beginning in the 1990s. In general, scholars have conducted two primary lines of research to investigate this topic. The first such line of research has focused primarily on analyzing and monitoring the distribution of PhD holders across various sectors of the labor market based on large-scale surveys or official employment statistics data (Ehrenberg, 1992; Kyvik & Olsen, 2012). These studies have revealed that the increasing tendency of PhD holders to take on nonacademic careers is consistent across countries. Nonetheless, these studies have faced criticism due to their failure to elucidate the reasons underlying the preferences or self-selection associated with different kinds of occupations by PhD students (Roach & Sauermann, 2010). Consequently, another line of research has been pursued to explore the factors that may influence the career intentions of PhD students or the actual career choices made by PhD holders. Specifically, these studies have focused almost exclusively on the roles played by demographic and academic factors such as gender (Shen et al., 2018), supervision style (Curtin et al., 2016), research training (X. Zheng et al., 2024), and scientific output (Shen et al., 2018) in efforts to predict PhD students’ career intentions.

The complex psychological mechanisms underlying doctoral students’ professional development have received limited attention. A few recent studies have began to address this complexity, using frameworks such as social cognitive career theory (Lee & Shin, 2024) and the theory of planned behavior (Evers & Sieverding, 2015). These and similar theories of professional development have been criticized by Antony and Schaps (2021) for primarily highlighting the fundamentals of internalization or adoption processes associated with specific professional norms, values, and ethics, thereby largely ignoring the agency of the individual and the interactions between individuals and the surrounding environment. The emerging perspective of career construction theory (CCT) posits that career development should be dominated by individuals rather than organizations in the modern professional world, in which context individuals choose employers or jobs according to their own career development goals (Savickas et al., 2009). From this perspective, the transition from doctoral education to employment should be viewed as the embodiment of students’ progress from self-organization on the basis of specific occupational norms to self-extension in accordance with their own career development plan, which reflects the individual’s process of career self-construction and the successful integration of individual needs with societal expectations (Savickas, 2013).

In light of the limitations and theoretical challenges of previous studies, this study explicitly explores the influences of proactive personality, employment social capital, career adaptability, and gender on Chinese PhD students’ research career intentions by employing an extended CCT framework. To enhance the explanatory capabilities of our research model, we integrate employment social capital into the CCT framework because the critical roles played by actual and potential social resources in efforts to promote PhD students’ career development have been repeatedly emphasized in the literature (St. Clair et al., 2019; Liu et al., 2020). Moreover, the employment issues faced by females with PhDs have always been a significant area of focus in doctoral education research and practice at the global level (Boulos, 2016). Gender has been repeatedly identified as a critical sociocultural factor influencing individuals’ professional socialization and development in the context of doctoral education (Gardner & Mendoza, 2010). Several studies have highlighted the gender gap in PhD students’ career intentions and the relevant influencing factors (Carriero et al., 2024; Huang & Shen, 2019; Shen et al., 2018). However, we must still seek additional evidence regarding this gender gap in the relationships among various contextual and psychological factors and PhD students’ research career intentions.

In summary, the primary aims of this study are to employ an extended CCT model to achieve the following: (1) examine the direct influence of proactive personality on PhD students’ research career intentions; (2) test the mediating effects of PhD students’ career self-construction process, namely, employment social capital and career adaptability, in connecting these students’ proactive personality with their research career intentions; and (3) explore the moderating role of gender in the relationships among proactive personality, employment social capital, career adaptability, and PhD students’ research career intentions. Accordingly, we contribute to the literature by introducing a self-construction perspective on PhD students’ research career intentions. Furthermore, by obtaining a holistic understanding of the factors that can enhance PhD students’ intentions to remain in research careers, this study can offer PhD-granting institutions and policy-makers practical recommendations that can guide the reform and development of an approach to doctoral education that seeks to encourage PhD students to pursue research careers.

## 2. Theoretical Background and Hypothesis Development

### 2.1. The Extended CCT

The CCT was formally proposed by Savickas in 2002, and it systematically integrates the philosophical views of individual constructivism, social constructivism, and postmodernism (Savickas, 2002). Adaptation is the core proposition of the CCT and focuses on the coping processes and strategies employed by individuals in the context of career development; that is, it enables individuals to transition smoothly among different roles or tasks (e.g., from school to work or from one job to another) and thus achieve a beneficial person–environment match (Savickas, 2013). The basic premise of the CCT is that effective career development involves a process of sequential adaptation that occurs through the harmonious fusion of personal needs with social expectations (Savickas & Porfeli, 2012). Specifically, the CCT posits that a variety of individual differences in traits (e.g., personal agency or personality) may lead people to incorporate their positive psychological resources (e.g., career adaptability) into their career aspirations or work roles more or less successfully (Savickas et al., 2009). Since its initial proposal, the CCT has been widely used in empirical studies seeking to investigate students’ transitions from education to career. However, to our knowledge, only three studies have employed the CCT to explain subjective academic career success and career adaptability among doctoral students (Guan et al., 2018; Mei et al., 2021; Owusu-Agyeman, 2021).

Although the CCT has been viewed as one of the most remarkable theories in the field of occupational psychology in the past 20 years, a recent systematic review reported that some research questions in this context remain unanswered and thus require further empirical elaboration (Rudolph et al., 2019). One major concern in this respect is that the CCT ignores the role of contextual factors in shaping the adaptation processes outlined in the theory itself to some extent, particularly with regard to the role of those factors in the development of individuals’ psychological resources, such as career adaptability. The CCT clearly indicates that it is critical for individuals to receive career resources that support their active attempts to develop their careers and respond effectively to relevant opportunities and challenges (Savickas & Porfeli, 2012). Employability social capital has been identified as a beneficial contextual factor that facilitates the acquisition of psychological resources and the achievement of early professional socialization among PhD students (Liu et al., 2020; Peeters et al., 2019; Weng, 2020). For example, by accessing and utilizing career-relevant external support, resources, networks, and information, PhD students can exhibit their personal qualities, thereby developing positive attitudes and effective strategies that can help them accomplish their career aspirations (Blackford, 2018). Accordingly, employability social capital helps PhD students navigate the diverse needs of industry and academic research careers more efficiently (St. Clair et al., 2017). On the basis of these perspectives, we incorporate employability social capital into the career construction model and explore the chain mediating effects of employability social capital and career adaptability on the relationship between proactive personality and research career intentions among PhD students.

**Figure 1:**
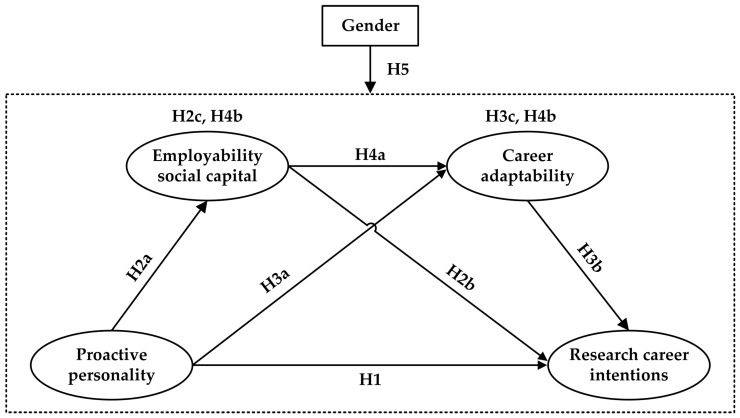
Research framework. Note: H2c, the indirect relationship between proactive personality and research career intentions via employability social capital; H3c, the indirect relationship between proactive personality and research career intentions via career adaptability; H4b, the indirect relationship between proactive personality and research career intentions via the chain mediation of employability social capital and career adaptability.

Moreover, regarding situations in which the CCT is used to explain individuals’ career intentions and choices, scholars have called for more attention to be given to specific and underrepresented groups, such as females, in research professions, thus extending this theory and identifying additional relevant boundary conditions (Rudolph et al., 2019). Although women, particularly those who have obtained doctoral degrees, account for a significant proportion of the research workforce, an increasing number of studies have reported that women may encounter a number of barriers to their career development that cause them to remain dramatically underrepresented in the upper echelons of academic and research institutions (Hüttges & Fay, 2015). To obtain deeper insights into this gender gap with respect to the relationships between various factors and research career intentions among PhD students, we further include gender as a moderating factor in this extended CCT model. Figure 1 illustrates the research framework for our study, which is based on the extended CCT.

### 2.2. Proactive Personality and Research Career Intentions

The concept of proactive personality was originally proposed by Bateman and Crant (Bateman & Crant, 1993) in their discussion of the proactive aspects of organizational behavior. These authors specified that proactive personality refers to the stable tendency of an individual to take the initiative to influence the surrounding environment. Specifically, proactive individuals are less constrained by the environment but rather actively change that environment; accordingly, such individuals can identify opportunities and take a series of proactive actions until they can make meaningful changes. In contrast, nonproactive individuals react passively to the environment, adapt negatively to the environment, or are even shaped by the environment; such individuals are thus unable to recognize opportunities, let alone use those opportunities to make a difference (Crant, 2000). Both theoretical and practical perspectives have suggested that individuals who exhibit high levels of proactive personality are more likely to form career goals and aspirations that are consistent with their primary capabilities and interests in the occupation that they seek to enter; thus, they can engage with a crowded labor market more effectively (Lent et al., 2000).

In this context, research career intentions are defined in terms of the extent to which PhD students tend to pursue (or not pursue) research-related careers in either academic or nonacademic settings, which are the most proximal antecedents of their actual research career choices, as suggested by the theory of planned behavior (Ajzen, 2002). Several empirical studies that have investigated higher education have reported that proactive students take self-reliant and self-directed actions to optimize their current circumstances and maintain strong aspirations with regard to their future employment, which in turn influences their career development outcomes. For example, Hsieh and Huang (2014) investigated a sample of 336 college students and reported that proactive personality is positively associated with career decision self-efficacy. Zhou et al. (2021) reported similar findings by reference to a sample of 743 Chinese university graduates, indicating that proactive personality positively influences students’ career decision-making self-efficacy. Moreover, Zhang et al. (2024) recently conducted a survey of 980 undergraduates from 16 universities in China; the findings revealed that proactive students are more active in career exploration activities. Although the relationship between proactive personality and career intentions among PhD students has yet to be explored in full detail, scholars have indicated that PhD students who exhibit high levels of proactive personality are able to identify both existing and potential career opportunities more effectively and to determine whether these opportunities match their own skills and needs more accurately and with minimal resource costs (Germain-Alamartine et al., 2021). On the basis of these theoretical perspectives and the findings of previous empirical research, we thus propose the following hypothesis:

**Hypothesis** **1** **(H1).** *Proactive personality is positively related to research career intentions among PhD students*.

### 2.3. The Mediating Role of Employability Social Capital

The social capital perspective may provide a useful argument regarding the mechanisms through which proactive PhD students develop and act upon research career intentions. Social capital is a crucial component of employability capital (Donald et al., 2024). Tomlinson (2017) defined employability social capital as “the sum of social relationships and networks that help mobilize graduates’ existing human capital and bring them closer the labor market and its opportunity structures” (p. 342). Individuals’ social relationships and networks determine the extent to which they can obtain access to various potential or actual employment resources (Coleman, 1988). The social capital perspective highlights the fact that proactive PhD students do not operate in a social vacuum (Bozeman et al., 2001; Liu et al., 2020). Rather, they must take proactive advantage of their first-hand research career experience, support, resources, and information both within and beyond the campus setting in their pursuit of self-directed career objectives and success (Seo & Yeo, 2020; Thompson, 2005). An increasing number of studies have demonstrated the close relationships among proactive personality, employability social capital, and career intentions. For example, one longitudinal study revealed that proactive personality is positively related to social capital and that the resource dimension and the relational dimension of social capital sequentially and positively mediate the relationship between personality and turnover intentions among 174 Chinese employees (Yang et al., 2011). In the context of higher education, Y. F. Luo et al. (2022) reported that proactive personality has a significant and positive influence on college students’ entrepreneurial intentions, whereas social capital mediates the relationship between these factors. On the basis of these theoretical arguments and empirical evidence, we argue that the establishment related to strong social relationships and networks represents a likely avenue through which proactive PhD students achieve high levels of performance. Hence, we propose the following hypotheses:

**Hypothesis** **2a** **(H2a).** *Proactive personality is positively related to employability social capital among PhD students*.

**Hypothesis** **2b** **(H2b).** *Employability social capital is positively related to research career intentions among PhD students*.

**Hypothesis** **2c** **(H2c).** *Employability social capital mediates the relationship between proactive personality and research career intentions among PhD students*.

### 2.4. The Mediating Role of Career Adaptability

As the core element and a so-called psychosocial construct in the CCT model, career adaptability reflects the resources or self-regulatory metacompetency that individuals require to cope successfully with both current and anticipated career-related tasks, transitions, and challenges (Savickas & Porfeli, 2012). Career theorists have posited that individuals who engage in self-directed practices, self-manage their career development activities confidently, and take control of the direction of their own careers can overcome challenges in the labor market more effectively and can obtain more advantageous career circumstances than individuals who are less engaged in such behaviors (Lent et al., 2016). In the field of higher education, studies have repeatedly demonstrated that career adaptability is one of the primary factors involved in students’ career decision making (Chen et al., 2022), academic persistence intentions (Wilkins-Yel et al., 2018), career exploration (Ma et al., 2023), and career intentions (Al-Waqfi et al., 2023). Based on the CCT, for example, Al-Waqfi et al. (2023) reported that career adaptability significantly influences college students’ willingness to work in the private sector. Moreover, career adaptability is likely to be developed more effectively by individuals who exhibit high levels of proactive personality (Tolentino et al., 2014), and empirical studies have provided evidence to support the mediating role of career adaptability in the relationship between proactive personality and individuals’ career development. For example, in a recent meta-analysis, Yu et al. (2023) revealed that career adaptability mediates the relationship between proactive personality and students’ subjective career success during the career exploration stage. Wang et al. (2021) also found that career adaptability mediated the relationship between proactive personality and career growth potential among 903 Chinese university students. Based on these theoretical perspectives and empirical findings, we agree with the argument that career adaptability may help proactive PhD students effectively navigate the increased uncertainty and competitiveness that characterize the doctoral labor market while continuing to insist on their desired career paths (Kvasková et al., 2023). Thus, we propose the following hypotheses:

**Hypothesis** **3a** **(H3a).** *Proactive personality is positively related to career adaptability among PhD students*.

**Hypothesis** **3b** **(H3b).** *Career adaptability is positively related to research career intentions among PhD students*.

**Hypothesis** **3c** **(H3c).** *Career adaptability mediates the relationship between proactive personality and research career intentions among PhD students*.

### 2.5. The Chain Mediating Roles of Employability Social Capital and Career Adaptability

As mentioned above, although strong connections among proactive personality, social capital, career adaptability, and individual career development variables have been well documented in the extant literature, minimal research has investigated the sequential mediating effects of social capital and career adaptability in association with proactive personality and individual career intentions. According to the CCT, the notion of proactive personality refers to the likelihood that PhD students will participate actively in research training and career development activities; thus, this factor can help them establish research career-related social networks and relationships (Mei et al., 2021), thereby encouraging them to develop the readiness and agency they need to interact with other individuals who can meet their research career needs and enable them to preserve their research career aspirations (Forbrig & Kuper, 2021). This account implies that employability social capital and career adaptability, which represent two important process and resource factors, may play crucial mediating roles in the relationship between proactive personality and research career intentions. In fact, previous empirical studies have provided valuable clues regarding the chain mediating roles of social capital and career adaptability in this context. For instance, by reference to 610 Chinese college students, Hu et al. (2021) reported that perceived social support mediates the relationship between proactive personality and career adaptability. Sou et al. (2022) revealed that career adaptability mediates the relationship between social capital and career engagement by investigating a sample of 610 university students in Macao. Therefore, we propose the following hypotheses:

**Hypothesis** **4a** **(H4a).** *Employability social capital is positively related to career adaptability among PhD students*.

**Hypothesis** **4b** **(H4b).** *Employability social capital and career adaptability play a chain mediating role in the relationship between proactive personality and research career intentions among PhD students*.

### 2.6. The Moderating Role of Gender

One advantage of the CCT over other career development theories is that the CCT explicitly incorporates individual background variables, including gender, into its empirical framework with the goal of explaining people’s career development and choices. The CCT posits that the early socialization experiences of individuals in their specific sociocultural environments have significant effects on their career development and outcomes (Savickas, 2013). Specifically, the male-dominated tradition and rapid expansion of doctoral education may exacerbate the already significant gender disparities regarding the ability to access essential resources within the scientific and research career ecosystem (Abramo et al., 2013; Bozzon et al., 2018). This situation may lead to variations in the ability of female PhD students to accumulate psychosocial resources (Williams & Subich, 2006). Female PhD students may address these issues of limited career resources, opportunities, and information by proactively establishing external social relationships and networks (Epstein & Lachmann, 2018; Fuchs et al., 2001; Taasoobshirazi & Carr, 2008). For example, with regard to a large research university in the USA, Moors et al. (2014) reported that the positive effects of perceived social support on job satisfaction and sense of belonging to the workplace were weaker among male STEMM (i.e., science, technology, engineering, math, and medical science) postdocs than among comparable female STEMM postdocs. In this sense, female PhD students might obtain greater psychological and behavioral advantages from different types of social capital than can their male counterparts. Moreover, female PhD students are also more likely to need psychosocial resources to strengthen their research career intentions than male students. For instance, Yiming et al. (2023) reported that self-efficacy seems to significantly contribute to career identity among female college students but not among male college students. On the basis of previous theories and research, we thus propose the following hypothesis:

**Hypothesis** **5** **(H5).** *Gender moderates the relationships among proactive personality, employability social capital, career self-efficacy, and research career intentions among PhD students such that these relationships are stronger among female PhD students than among male PhD students*.

## 3. Methodology

### 3.1. Participants and Procedure

The data used in this study were drawn from the professional development module of the 2023 Doctoral Students Experience Survey at 10 “Double First-Class” universities in Beijing and Xi’an, China. This survey was designed and implemented by the Postgraduate Education Research Center and the Higher Engineering Education Research Center at Beihang University in 2023, specifically during the autumn semester of the 2023–2024 academic year. The questionnaires were administered to the target doctoral students via a professional online survey platform (www.sojump.com) with the assistance of the postgraduate administration departments of the universities that participated in the survey.

The instruction page of the questionnaire provided respondents with information regarding the purpose of the study, the anonymity and confidentiality of the survey, and other issues that they were required to be aware to ensure that they could respond to the survey items in a valid manner. A total of 836 questionnaires were returned. To ensure the quality of our data, we removed 41 participants who exhibited straight-line response patterns (Zhang & Conrad, 2014). Ultimately, 795 valid responses were retained for the subsequent data analysis. No significant differences in the demographic variables were observed between the final sample and the original sample in terms of the results of the chi-square tests (*p* > 0.05). The average age of the respondents was 26.70 years (SD = 3.12), and Table 1 presents the basic demographic information for the final sample.

### 3.2. Measurement

The questionnaire designed for this study consisted primarily of two sections. In the first section, we collected the respondents’ demographic information. The second section presented respondents with the scales used to measure the four primary variables under investigation. These four scales and the corresponding items included in this study were all drawn from established scales that have been published in peer-reviewed journals and verified by empirical studies across different contexts and cultures.

Research career intentions were evaluated via four items that were drawn from previous studies (Bramlage et al., 2021; Liñán & Chen, 2009) and adapted to suit the context of doctoral education. The PhD student respondents rated each item on a five-point Likert scale ranging from 1 (strongly disagree) to 5 (strongly agree); higher scores indicated stronger intentions to pursue a research career.

Proactive personality was assessed via three items that were drawn from Shang and Gan (2009), who adapted this measurement from Bateman and Crant (1993) to suit the context of higher education in China. The proactive personality scale is used to assess an individual’s stable disposition toward the notion of engaging in proactive behaviors with the goal of affecting their surroundings without substantial concern for situational challenges. The items included in this measure were rated on a five-point Likert scale ranging from 1 (strongly disagree) to 5 (strongly agree).

A four-item employability social capital scale was derived from studies conducted by Eby et al. (2003) and Hirschi et al. (2018). We asked the PhD students included in this research to indicate their level of agreement with various statements regarding their career networking and the career support they received on a 5-point Likert scale ranging from 1 (strongly disagree) to 5 (strongly agree).

Career adaptability was measured via five items that were adapted from the Chinese version of the career adaptability scale (Hou et al., 2012; Savickas & Porfeli, 2012). We required the PhD students to indicate how strongly they had obtained resources to support their career development on a 5-point Likert scale ranging from 1 (strongly disagree) to 5 (strongly agree).

As indicated in Table 2, the mean values of the scale items varied from 3.108 to 4.169, the standard deviations varied from 0.841 to 1.069, the absolute coefficients of skewness ranged between 0.680 and 1.507, i.e., lower than the threshold level of 3, and the absolute coefficients of kurtosis ranged between 0.051 and 2.352, i.e., lower than the threshold level of 10; thus, these findings indicate that the measurement items followed approximately normal distributions and that the subsequent analysis could be thus be performed (Hair et al., 2010). Moreover, the Cronbach’s α coefficients for the four constructs varied from 0.898 to 0.950, i.e., higher than the recommended value of 0.700 (Nunnally, 1978).

### 3.3. Data Analysis

We followed the two-step procedure suggested by Anderson and Gerbing (1988) by first conducting a series of confirmatory factor analyses (CFAs) with the goal of detecting the reliability and validity of a measurement model consisting of the four focal variables before we tested the hypotheses. Then, we performed structural equation modeling (SEM) with latent variables to examine the relationships hypothesized in this study in further detail. In addition, given that the data were collected simultaneously from a single source, we conducted Harman’s single-factor test with the goal of detecting potential common method bias (Podsakoff et al., 2003). We used standard fit indices, including the ratio of chi-square to the degree of freedom (*χ*^2^/*df* < 5), goodness-of-fit index (GFI > 0.90), incremental fit index (IFI > 0.90), comparative fix index (CFI > 0.90), Tucker–Lewis index (TLI > 0.90), standardized root mean square residual (SRMR < 0.08), and root mean square error of approximation (RMSEA < 0.08), to evaluate the goodness-of-fit exhibited by the models (Kline, 2011). To investigate the potential mediating effects included in the research model, we further conducted a bootstrapping analysis based on 5000 random resamples with the aim of providing bias-corrected estimates of a series of indirect effects of proactive personality on research career intentions alongside the corresponding 95% confidence intervals (CIs, two-tailed). When a 95% CI does not contain zero, the corresponding mediating effect is statistically significant at the 0.05 level (Preacher & Hayes, 2008). Moreover, we performed a multigroup SEM analysis to test the possible moderating effects of gender on the structural relationships. All these statistical analyses were conducted with the assistance of the Amos 26 software package.

## 4. Results

### 4.1. Measurement Model

**Figure 2:**
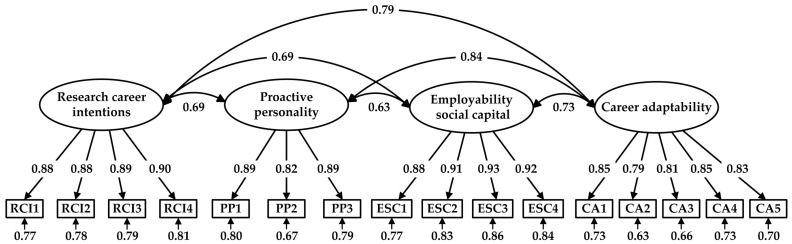
Results of the CFA.

We conducted a series of CFAs to confirm the fit of the four-factor measurement model to the data, as well as the distinctiveness of the focal variables included in this study. The measurement model consisted of four latent constructs and 16 observed indicators. In the CFA, latent constructs were allowed to be freely correlated with each other, whereas the observed indicators were specified to load only on the corresponding latent constructs. The results of the CFA revealed that the measurement model exhibited a good fit to the data (*χ*^2^ = 479.746; *df* = 98; *χ*^2^/*df* = 4.895; GFI = 0.923; CFI = 0.969; IFI = 0.969; TLI = 0.962; SRMR = 0.030; RMSEA = 0.070 [90% CI: 0.064, 0.076]). As illustrated in Figure 2, all standardized item loadings were significant and greater than the threshold of 0.50 (Kline, 2011).

We then compared the measurement model (i.e., the four-factor model) with three alternative models (i.e., models containing one to three factors). As presented in Table 3, the fit indices of all the alternative models failed to meet the recommended criteria (Kline, 2011). These findings support the discriminant validity of the four latent variables under analysis in this research. Moreover, to detect potential common method bias in the research data, we compared the measurement model with the model including a common method factor (i.e., the five-factor model). As presented in Table 3, although the five-factor model improved the goodness-of-fit index over the hypothesized four-factor model (Δ*χ*^2^ [Δ*df* = 2] = 58.434, *p* < 0.001), in the comparison between these two models, the changes in the CFI and RMSEA values were only 0.006 and 0.001, respectively; these changes did not exceed the suggested rule of thumb of 0.05 (Bagozzi & Yi, 1990), thus indicating that common method bias did not seriously affect our results.

Following the CFA, the composite reliability (CR), average variance extracted (AVE), and correlation coefficients of each construct were calculated. As presented in Table 4, the CR values of each variable were all above the standard threshold of 0.70, and the AVE values also satisfied the criterion of 0.60. Additionally, significant correlations were observed among the focal variables, and the square root of the AVE was greater than the correlation coefficients between each pair of variables. These results indicate that the measures included in this study exhibited excellent convergent and discriminant validity (Fornell & Larcker, 1981).

### 4.2. Structural Model

**Figure 3:**
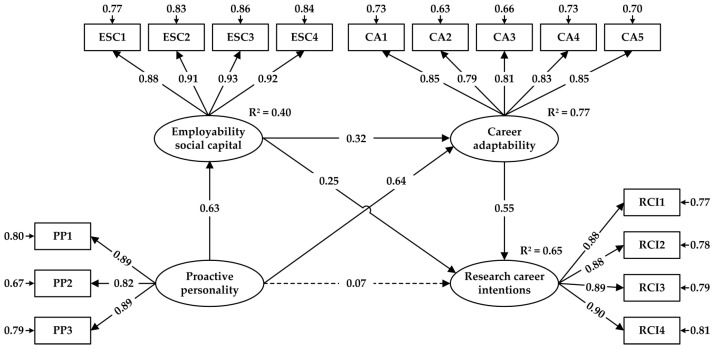
Results of the SEM analysis.

We conducted a structural equation model analysis with the goal of testing the hypothesized relationships among the variables. The structural model exhibited a good fit to the data: *χ*^2^ = 479.746; *df* = 98; *χ*^2^/*df* = 4.895; GFI = 0.923; CFI = 0.969; IFI = 0.969; TLI = 0.962; SRMR = 0.030; RMSEA = 0.070 [90% CI: 0.064, 0.076]. As illustrated in Figure 3, the direct link between proactive personality and research career intentions was not significant (*β* = 0.069, *SE* = 0.062, *p* > 0.05). Thus, H1 was not supported. In line with our expectations, we found that proactive personality had a significant positive relationship with employability social capital (*β* = 0.631, *SE* = 0.039, *p* < 0.001), which in turn was positively related to the research career intentions of PhD students (*β* = 0.252, *SE* = 0.038, *p* < 0.001). Therefore, H2a and H2b were supported. Similarly, proactive personality was also positively related to career adaptability (*β* = 0.639, *SE* = 0.034, *p* < 0.001), which in turn was positively associated with the research career intentions of PhD students (*β* = 0.546, *SE* = 0.074, *p* < 0.001). Therefore, H3a and H3b were supported. In addition, the relationship between employability social capital and career adaptability was significant (*β* = 0.323, *SE* = 0.027, *p* < 0.001), thereby supporting H4a.

We further tested the mediating effects included in the structural model by conducting bootstrapping analysis. As indicated in Table 5, the indirect effects of proactive personality on research career intentions via employability social capital (indirect effect = 0.177, *SE* = 0.044, 95% CI [0.097, 0.268]) and career adaptability (indirect effect = 0.387, *SE* = 0.068, 95% CI [0.265, 0.534]) were both significant, thereby supporting H2c and H3c. Furthermore, the chain mediating roles of employability social capital and career adaptability in the relationship between proactive personality and research career intentions among PhD students were confirmed (indirect effect = 0.123, *SE* = 0.026, 95% CI [0.078, 0.181]), thus supporting H4b.

### 4.3. Moderating Effects

A multigroup SEM analysis was conducted to test the moderating effects of gender on the structural relationships included in the proposed model. Specifically, the first step in this process involved examining the difference between an unconstrained model (which allowed for free estimation of the paths among the variables) and a constrained model (which required all paths among the variables to be equal). In both models, factor loadings were held constant to ensure that the testing structure was the same between the male and female groups; however, the error terms were allowed to be estimated freely in both models. If a significant difference in the *χ*^2^ values was observed between the two models, such a finding would indicate that gender had a moderating effect on one or more paths (Wu, 2013). The *χ*^2^ difference test revealed that the constrained model (*χ*^2^ = 729.919, *df* = 214) and the unconstrained model (*χ*^2^ = 714.322, *df* = 208) exhibited significant differences (Δ*χ*^2^ = 15.597, Δ*df* = 6, *p* < 0.05), thus supporting the moderating role of gender in the proposed model.

To detect the moderating effects of gender on specific paths more carefully, a series of *χ*^2^ difference tests were performed to investigate the constrained model and five unconstrained models (each of which allowed only the path being tested to be estimated freely) (Augustus-Horvath & Tylka, 2009). As presented in Table 6, gender significantly moderated only one out of the six paths within the model. Specifically, we found that the impact of employability social capital on research career intentions was stronger among female PhD students (*β* = 0.322, *SE* = 0.048, *p* < 0.001) than among male PhD students (*β* = 0.212, *SE* = 0.042, *p* < 0.001). Therefore, H5 was partially supported.

## 5. Discussion and Implications

The aim of this study was to explore the factors influencing the research career intentions of Chinese PhD students on the basis of an extended CCT framework. Specifically, we examined the relationship between proactive personality and research career intentions alongside the mediating effects of employability social capital and career adaptability on this relationship and the moderating role of gender in the proposed model. The main findings of this research are as follows.

First, contrary to our expectations, this study revealed that proactive personality has no significant direct effect on PhD students’ research career intentions. This finding indicates that high levels of proactive personality on the part of PhD students do not necessarily result in increased intentions to pursue research careers. This finding may be explained by the fact that although the diversified employment of PhD graduates and holders has become the “new normal” in the PhD labor market, the process of cultivating researchers, especially those working in the academic sector, is still viewed by policy-makers, PhD-granting institutions, and supervisors as the primary value and goal of PhD education and the PhD degree itself (G. Zheng et al., 2018). Therefore, PhD students and their supervisors may misperceive each other’s goals and desires, thus making it difficult for PhD students to access the support they need for their career development from their supervisors and the academic environment. Nonetheless, proactive PhD students may be less affected by the challenges surrounding them and thus tend to set their own career goals and to engage in career exploration and preparation behaviors independent of the powerful norms and expectations of their supervisors and institutions (Crant, 2000). Thus, this finding highlights the necessity of exploring the complex process leading from individual personality characteristics to career outcomes.

Second, we found that both employability social capital and career adaptability mediate the relationship between proactive personality and research career intentions. With respect to employability social capital, this study supports the claim that PhD students who exhibit higher levels of proactive personality are more likely to take full advantage of first-hand research experience, support, resources, and information both within and beyond the campus setting in the process of establishing their research career objectives (Seo & Yeo, 2020). The basic components of employability social capital are career-related social relationships and networks. This finding implies that PhD students who exhibit high levels of proactive personality are more likely to seek ways of constructing a social environment and actively strive to attach themselves to people who occupy positions of influence and power, a situation which is conducive to their own job success (Thompson, 2005). With respect to career adaptability, which represents a core construct in the CCT, previous studies have reported that individuals who exhibit high levels of proactivity are more likely to obtain and develop their adaptability resources and experience a resource gain spiral, thus leading to better career adaptation results (Wang et al., 2021; Yu et al., 2023). Our finding further supports this conclusion. High levels of career adaptability can increase PhD students’ competencies in the context of career decision making and help them adopt adaptive strategies to navigate their career paths and ultimately achieve their career goals (Kvasková et al., 2023).

Third, consistent with our expectations, we revealed that employability social capital and career adaptability have a significant chain mediating effect on the relationship between proactive personality and research career intentions among PhD students. In combination with the nonsignificant path between proactive personality and research career intentions among PhD students, the chain mediating effect observed in this study may provide new evidence that can expand our understanding of the mediating mechanisms underlying the relationship between proactive personality and career outcomes (Hu et al., 2021; Sou et al., 2022). Personality constructs may be too general to predict specific career intentions or career choices. Thus, PhD students who exhibit high levels of proactive personality generally tend to shape and take advantage of broader research networks and relationships in a self-directed manner, which may lead to a high level of research career intentions by promoting the flourishing of internal psychosocial resources and competences.

Fourth, we highlighted the moderating role of gender in the relationship between employability social capital and research career intentions. Specifically, female PhD students were associated with stronger positive associations between employability social capital and research career intentions than male students. This result is in line with the findings of previous studies that have reported that social relationships, networks, guidance, and support are more valuable for females than for males in terms of their decisions regarding whether to adopt research careers (Taasoobshirazi & Carr, 2008). According to Epstein and Lachmann (2018), female PhD students who exhibit deficient social capital might be more vulnerable to employment problems than males. Therefore, female PhD students may rely more on social relationships and networks to develop or maintain their research career intentions than male PhD students do. In contrast, male PhD students may access and obtain resources that can support their research career intentions from broader and more convenient sources because of conventional gender expectations and the advantages available to males in the world of professional research (Yuan et al., 2024).

The findings of this research have several important theoretical and practical implications. Theoretically, although the literature has consistently highlighted the importance of psychosocial constructs with regard to the professional socialization process of PhD students (Guan et al., 2018; Mei et al., 2021; Owusu-Agyeman, 2021), the potential roles of employment-related contextual factors have largely been neglected. On the basis of an expanded CCT framework, our study bridges this gap by emphasizing the significance of employability social capital as an explanation of career development among PhD students. The results of this research expand our understanding of the complex mediating mechanisms involved in the development of research career intentions among PhD students who exhibit high levels of proactive personality. We further integrate gender as a moderator into the expanded CCT model, thus offering a more nuanced understanding of the gender-related conditions associated with the relationship between social capital and research career intentions in the context of doctoral education. Moreover, the moderating role of gender observed in this study enriches the literature on gender effects and issues within male-dominated research professions to some extent (Sáinz et al., 2016).

Higher education institutions and industrial R&D departments seek to attract and retain researchers with PhDs with the goal of increasing their scientific, technological, and innovation competitiveness and excellence in the context of a society based on the knowledge economy (Dorenkamp & Weiß, 2018). The practical guidance offered by this study with regard to enhancing PhD students’ research career intentions lies in its identification of the chain mediating roles of employability social capital and career adaptability and the moderating role of gender in this context. First, the nonsignificant direct path observed in this study does not indicate that proactive personality is unimportant with regard to efforts to promote students’ research career intentions. Instead, doctoral education should not only help PhD students acquire theoretical knowledge and research skills but also focus on cultivating their proactivity, which may help students actively access resources and develop the competences they need to pursue a research career. Second, we recommend that policy-makers should attach more importance to the development and accumulation of employability resources on the part of PhD students. For example, institutions should actively initiate programs and mentorships with the goal of helping connect trainees to alumni and professionals working in broad research areas, thereby encouraging PhD students to seek career resources and opportunities proactively as well as increasing their adaptability in contexts in which the doctoral labor market is characterized by increased uncertainty, precariousness, and competitiveness. Finally, institutions and supervisors should note that career-related social capital appears to be particularly critical with regard to efforts to enhance female PhD students’ research career intentions. Therefore, it is vital to develop more inclusive and interactive career guidance projects with the aim of helping female PhD students establish more social relationships and networks that are conducive to their research careers.

## 6. Conclusions and Directions for Future Research

In summary, on the basis of an expanded CCT framework, this study provides empirical evidence regarding the structural relationships among proactive personality, employability social capital, career adaptability, and PhD students’ research career intentions, as well as the moderating role of gender in these relationships. Therefore, the CCT may serve as a useful meta-framework for understanding the career development process and the corresponding outcomes among PhD students. However, several limitations of this study should be noted and addressed in future research. This study used a self-report survey and featured a cross-sectional study design, thus leaving room for further exploration concerning the causal relationships among relevant variables based on longitudinal designs and mixed-method (i.e., quantitative with qualitative) approaches. Furthermore, future research should investigate in further detail the influence of other critical contextual and psychological factors on PhD students’ research career development and outcomes as well as the potential mechanisms and boundary conditions underlying the relationships among these factors on the basis of the CTT framework.

## Figures and Tables

**Table 1:** Demographic information regarding the doctoral student respondents (*N* = 795).

Variable	Group	*n*	Percentage (%)
Gender	Female	301	37.86
	Male	494	62.14
Major	Science and engineering	568	71.45
	Humanities and social sciences	227	28.55
Grade	First year	174	21.89
	Second year	183	23.02
	Third year	166	20.88
	Fourth year or above	272	34.21

**Table 2:** Measurement items and descriptive statistics.

Variables/Measurement Items	Mean	SD	Skewness	Kurtosis
**Research career intentions (RCI, Cronbach’s α = 0.934)**				
RCI1: A research career is attractive to me.	3.108	0.971	−0.971	0.468
RCI2: I have very seriously considered working as a researcher.	3.148	1.014	−1.180	0.901
RCI3: A research career within or outside academia would bring me great satisfaction.	3.355	0.858	−1.471	2.228
RCI4: I plan to pursue a research career after earning my doctoral degree.	3.364	0.862	−1.507	2.352
**Proactive personality (PP, Cronbach’s α = 0.898)**				
PP1: No matter what the odds are, if I believe in something, I will make it happen.	4.084	0.870	−0.761	0.376
PP2: I love being a champion for my ideas even in the face of opposition from others.	4.042	0.907	−0.812	0.509
PP3: I am excellent at identifying opportunities.	4.093	0.864	−0.802	0.459
**Employability social capital (ESC, Cronbach’s α = 0.950)**				
ESC1: I always try to be well connected in the professional field to which I aspire.	4.116	0.988	−1.066	0.721
ESC2: I frequently make contacts with other people who are important with regard to my career development.	3.941	1.052	−0.872	0.251
ESC3: My fellow students/supervisors support me in terms of my career development.	3.950	1.069	−0.841	0.051
ESC4: I receive a high level of career support from my social environment.	3.962	1.040	−0.840	0.133
**Career adaptability (CA, Cronbach’s α = 0.914)**				
CA1: Preparing for my future career.	4.169	0.888	−0.954	0.615
CA2: Becoming aware of the vocational choices that I must make.	3.906	1.015	−0.680	−0.064
CA3: Making career decisions by myself.	4.140	0.922	−1.004	0.755
CA4: Investigating options before making a career choice.	4.130	0.841	−0.720	0.118
CA5: Learning new skills in my field of research.	4.064	0.865	−0.686	0.149

**Table 3:** Results of the model comparisons.

Models	χ^2^ (*df*)	χ^2^/*df*	CFI	RMSEA
One-factor (abcd)	3390.831 (104)	32.604	0.735	0.200
Two-factor (abc-d)	2468.395 (103)	23.965	0.809	0.170
Three-factor (a-bc-d)	1907.223 (101)	18.883	0.854	0.150
Four-factor (a-b-c-d)	479.746 (98)	4.895	0.969	0.070
Five-factor (a-b-c-d-e)	418.407 (96)	4.358	0.974	0.065

Note: a = proactive personality; b = employability social capital; c = career adaptability; d = research career intentions; e = common method factor.

**Table 4:** Means, standard deviations, reliability, validity, and correlations among variables.

Variables	M	SD	CR	AVE	1	2	3	4	5	6	7
1. Gender	0.621	0.485	—	—	—						
2. Age	26.698	3.129	—	—	0.032	—					
3. Grade	2.674	1.159	—	—	−0.006	0.287 ***	—				
4. PP	4.073	0.803	0.900	0.751	0.014	−0.005	0.030	***0.*** ***867***			
5. ESC	3.992	0.968	0.951	0.828	−0.028	0.010	−0.046	0.585 ***	***0.*** ***910***		
6. CA	4.081	0.783	0.917	0.687	−0.054	−0.009	−0.042	0.766 ***	0.682 ***	***0.*** ***829***	
7. RCI	3.244	0.849	0.937	0.787	−0.016	0.014	−0.044	0.634 ***	0.656 ***	0.733 ***	***0.*** ***887***

Note: PP = proactive personality; RCI = research career intentions; ESC = employability social capital; CA = career adaptability. The diagonal elements (in bold italics) indicate the square roots of the AVE values; *** *p* < 0.001.

**Table 5:** Results of the bootstrapping analysis.

Paths	Bootstrapping		95% Bias-Corrected CI	
	Effect	Boot S. E.	Boot LLCI	Boot ULCI
PP → RCI	0.069	0.070	−0.070	0.200
PP → ESC	0.631 ***	0.030	0.569	0.687
PP → CA	0.639 ***	0.037	0.566	0.711
ESC → CA	0.323 ***	0.040	0.245	0.399
ESC → RCI	0.252 ***	0.059	0.140	0.369
CA → RCI	0.546 ***	0.088	0.369	0.715
PP → ESC → RCI	0.177 ***	0.044	0.097	0.268
PP → CA → RCI	0.387 ***	0.068	0.265	0.534
PP → ESC → CA → RCI	0.123 ***	0.026	0.078	0.181

Note: PP = proactive personality; RCI = research career intentions; ESC = employability social capital; CA = career adaptability; LLCI = lower level confidence interval; ULCI = upper level confidence interval. *** *p* < 0.001.

**Table 6:** Results of the multigroup SEM analysis.

	Standardized Coefficients		*χ*^2^ (*df*)	Δ*χ*^2^ (∆*df*)
	Female	Male		
**Constrained Model**	**-**	**-**	729.919 (214)	**-**
PP → ESC	0.627 **	0.634 ***	729.817 (213)	0.102
PP → CA	0.616 ***	0.655 ***	726.999 (213)	2.920
PP → RCI	0.072	0.050	729.695 (213)	0.224
ESC → CA	0.330 ***	0.317 ***	729.908 (213)	0.011
ESC → RCI	0.322 ***	0.212 ***	722.536 (213)	7.383 **
CA → RCI	0.566 ***	0.548 ***	729.576 (213)	1.343

Note: PP = proactive personality; RCI = research career intentions; ESC = employability social capital; CA = career adaptability. ** *p* < 0.01. *** *p* < 0.001.

## Data Availability

The data presented in this study are available on request from the corresponding author due to privacy because participants agreed to participate in the study but they did not give explicit and written consent for their data to be shared publicly.
